# Highly pathogenic avian influenza A/Guangdong/17SF003/2016 is immunogenic and induces cross-protection against antigenically divergent H7N9 viruses

**DOI:** 10.1038/s41541-021-00295-7

**Published:** 2021-02-26

**Authors:** Peter Radvak, Martina Kosikova, Yuan-Chia Kuo, Xing Li, Richard Garner, Falko Schmeisser, Ivan Kosik, Zhiping Ye, Jerry P. Weir, Jonathan W. Yewdell, Hang Xie

**Affiliations:** 1 Laboratory of Pediatric and Respiratory Viral Diseases, Division of Viral Products, Office of Vaccines Research and Review, Center for Biologics Evaluation and Research, United States Food and Drug Administration, Silver Spring, MD USA; 2 Laboratory of DNA Viruses, Division of Viral Products, Office of Vaccines Research and Review, Center for Biologics Evaluation and Research, United States Food and Drug Administration, Silver Spring, MD USA; 3 Laboratory of Viral Diseases, National Institute of Allergy and Infectious Diseases, National Institutes of Health, Bethesda, MD USA

**Keywords:** Adjuvants, Protein vaccines, Inactivated vaccines, Influenza virus, Influenza virus

## Abstract

Avian influenza A(H7N9) epidemics have a fatality rate of approximately 40%. Previous studies reported that low pathogenic avian influenza (LPAI)-derived candidate vaccine viruses (CVVs) are poorly immunogenic. Here, we assess the immunogenicity and efficacy of a highly pathogenic avian influenza (HPAI) A/Guangdong/17SF003/2016 (GD/16)-extracted hemagglutinin (eHA) vaccine. GD/16 eHA induces robust H7-specific antibody responses in mice with a marked adjuvant antigen-sparing effect. Mice immunized with adjuvanted GD/16 eHA are protected from the lethal LPAI and HPAI H7N9 challenges, in stark contrast to low antibody titers and high mortality in mice receiving adjuvanted LPAI H7 eHAs. The protection correlates well with the magnitude of the H7-specific antibody response (IgG and microneutralization) or HA group 2 stem-specific IgG. Inclusion of adjuvanted GD/16 eHA in heterologous prime-boost improves the immunogenicity and protection of LPAI H7 HAs in mice. Our findings support the inclusion of GD/16-derived CVV in the pandemic preparedness vaccine stockpile.

## Introduction

Since the first known human infection with H7N9 avian influenza A virus was detected in 2013, a total of 1568 laboratory-H7N9-related human infections, resulting in 615 deaths, were reported to the World Health Organization as of November 2020^1^. Most H7N9 infections occurred in older individuals (>60 years old) with underlying comorbidities who had close contact with infected poultry^2^. Patients primarily developed lower respiratory infections with severity ranging from viral pneumonia to respiratory failure^3,4^. Critically, the overall H7N9-related fatality rate is approximately 40%^5^, which is significantly higher than those caused by seasonal influenza or 2009 H1N1 pandemic^6^. Most H7N9 clinical isolates, e.g. A/Anhui/1/2013 (AH/1) and A/Shanghai/2/2013 (SH/2) from the first wave, or A/Hong Kong/125/2017 (HK/125) from the fifth wave, are low pathogenic avian influenza (LPAI) viruses, and contain only a monobasic cleavage site in hemagglutinin (HA)^7^. A highly pathogenic avian influenza (HPAI) subclade bearing a multibasic HA cleavage site emerged during the fifth wave after viruses evolved into several phylogenetically divergent HA groups^7,8^. These HPAI H7N9 viruses cause systemic infections with expanded tissue tropism and show enhanced virulence in humans, poultry, and experimental animals^8–11^. From October 2016 to September 2017, LPAI and HPAI H7N9 viruses co-circulated in Asia, resulting in 766 confirmed human infections with 288 deaths, making the fifth wave the most deadly A(H7N9) epidemic to date^12^.

In general, H7N9 viruses are inefficient in droplet transmission, though readily spread by contact transmission in cohoused ferrets^11^. However, a recent study reports that HPAI A/Guangdong/17SF003/2016 (GD/16) H7N9 virus isolated during the fifth wave infected ferrets via respiratory droplets without adaptation^13^, which supports the potential of HPAI H7N9 viruses for sustained human-to-human respiratory transmission. This poses a significant threat to public health, highlighting the urgency of developing effective H7N9 vaccines.

The development of H7N9 vaccines has mainly focused on LPAI virus-derived candidate vaccine viruses (CVVs). However, HPAI and LPAI viruses are antigenically distinct; ferret antisera raised by infection of LPAI virus-derived CVVs reacted poorly against HPAI viruses^12^. Thus, vaccines that can provide broad cross-protection against both LPAI and HPAI H7N9 viruses are highly desired. In this study, we compared the immunogenicity and cross-protection in mice immunized with HA derived from HPAI GD/16 of the fifth wave vs HA derived from LPAI H7N9 viruses. Our results indicate that GD/16-derived HA is more immunogenic than LPAI H7N9-derived HA and protects immunized mice against lethal challenges of LPAI and HPAI H7N9 viruses.

## Results

### Immunization of adjuvanted GD/16-extracted HA induces cross-reactive antibodies against LPAI H7N9 challenges

**Fig. 1:**
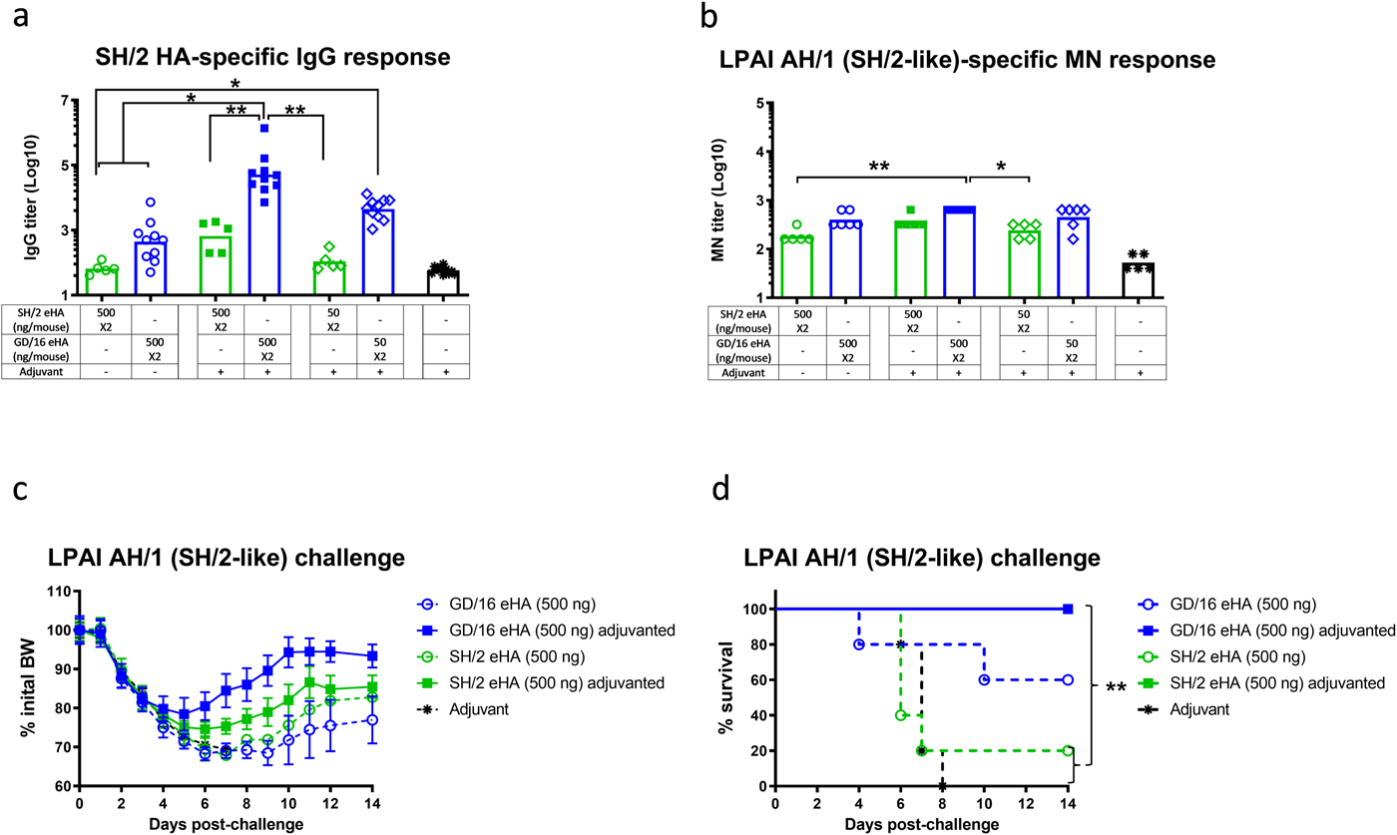
Immunogenicity and protection against low pathogenic avian influenza H7N9 virus of the first wave. BALB/c mice were immunized twice with HA extracted (eHA) from H7N9 candidate vaccine viruses A/Shanghai/2/2013 (SH/2) or A/Guangdong/17SF003/2016 (GD/16) with or without adjuvant. Immunized mice were challenged with low pathogenic avian influenza (LPAI) wild type (wt) H7N9 A/Anhui/1/2013 (AH/1, SH/2-like) of the first wave in a BSL-3+ biocontainment. (**a**) IgG ELISA titers against SH/2 HA; (**b**) microneutralization (MN) titers against LPAI wt AH/1 virus; (**c**) body weight (BW) and (**d**) survival after LPAI wt AH/1 challenge. Individual titers with geometric mean (bars) are shown (*n* = 5–10 mice/group). BW data are expressed as mean ± s.e.m (*n* = 5 mice/group). *p < 0.05 and **p < 0.01 by Kruskal-Wallis test with Dunn’s multiple comparisons after log transformation (antibody responses) or by Log-rank (Mantel-Cox) test (survival curves).

HAs extracted (eHAs) from formalin-inactivated H7N9 CVVs (Supplemental Fig. 1a, b) were further purified to minimize contamination with other viral proteins (Supplemental Fig. 1c). eHAs were then used to immunize mice with or without AddaVax™—a squalene-based adjuvant, similar to MF59 adjuvant included in FLUAD^®^ influenza vaccine. Compared to SH/2 eHA, immunization of mice with GD/16 eHA with or without adjuvant generally induced higher geometric mean titers (GMTs) of IgG against SH/2 HA by ELISA and microneutralization (MN) antibodies against LPAI wild type (wt) AH/1 virus (a SH/2-like strain) (Fig. 1a, b). Administration of 500 ng/mouse of adjuvanted GD/16 eHA induced the highest SH/2 cross-reactive IgG ELISA and MN GMTs among all doses tested (Fig. 1a, b). Mice receiving 50 ng adjuvanted GD/16 eHA also developed higher SH/2-specific IgG ELISA titers than those immunized with 500 ng of SH/2 eHA alone (*p* < 0.05) or with adjuvant (Fig. 1a). These data suggest that GD/16 eHA is more immunogenic than SH/2 eHA and induces a cross-reactive antibody response to SH/2. Following a lethal LPAI challenge with wt AH/1 (a SH/2-like virus), mice immunized with 500 ng unadjuvanted SH/2 or GD/16 eHA showed up to a 30% body weight (BW) loss and also 80% and 40% mortality, respectively (Fig. 1c, d). In contrast, immunization with 500 ng adjuvanted SH/2 or GD/16 eHA protected all mice against the lethal AH/1 challenge, and mice receiving adjuvanted GD/16 eHA showed less morbidity and recovered faster (Fig. 1c, d).

**Fig. 2:**
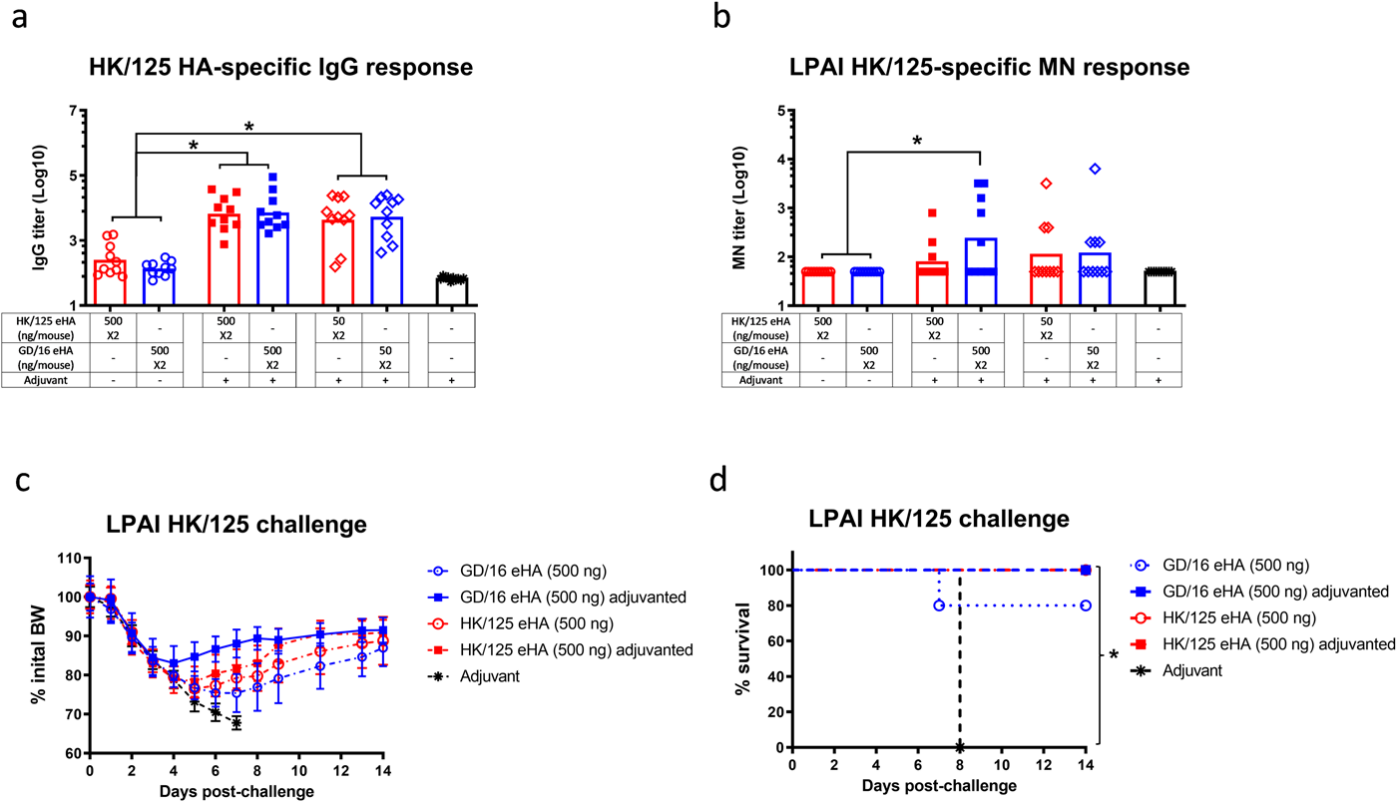
Immunogenicity and protection against Low pathogenic avian influenza H7N9 virus of the fifth wave. BALB/c mice were immunized twice with HA extracted (eHA) from H7N9 candidate vaccine viruses A/Hong Kong/125/2017 (HK/125) or A/Guangdong/17SF003/2016 (GD/16) with or without adjuvant. Immunized mice were challenged with low pathogenic avian influenza (LPAI) wild type (wt) H7N9 HK/125 virus in a BSL-3+ biocontainment. (**a**) IgG ELISA titers against HK/125 HA; (**b**) microneutralization (MN) titers against LPAI wt HK/125 virus; (**c**) body weight (BW) and (**d**) survival after LPAI wt HK/125 challenge. Individual titers with geometric mean (bars) are shown (*n* = 5–10 mice/group). BW data are expressed as mean ± s.e.m (*n* = 5 mice/group). *p < 0.05 by Kruskal–Wallis test with Dunn’s multiple comparisons after log transformation (antibody responses) or by Log-rank (Mantel–Cox) test (survival curves).

When compared to HK/125 eHA-immunized mice, immunization with GD/16 eHA at the same dose generally induced comparable levels of IgG ELISA titers against HK/125 HA and MN antibodies against wt HK/125 virus (Fig. 2a, b). Mice receiving 50 ng adjuvanted GD/16 and HK/125 eHAs showed significantly higher HK/125-specific IgG ELISA titers than the groups given 500 ng unadjuvanted eHAs, indicating an antigen-sparing effect by adjuvant (Fig. 2a). Consistent with the antibody responses, mice immunized with 500 ng GD/16 or HK/125 eHA with or without adjuvant showed similar morbidity and had 80–100% survival after a lethal LPAI wt HK/125 challenge (Fig. 2c, d).

Taken together, these findings indicate that GD/16 eHA is immunogenic and induces protective immunity in mice against lethal challenges of both LPAI AH/1 and HK/125 viruses from the first and fifth wave of human H7N9 viruses.

### Homologous prime-boost of adjuvanted GD/16 eHA protects mice from lethal HPAI H7N9 challenges

**Fig. 3:**
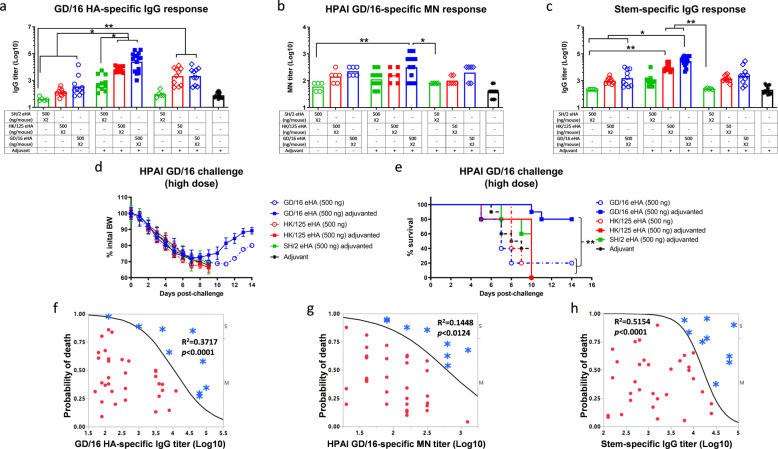
Immunogenicity and protection against highly pathogenic avian influenza H7N9 virus of the fifth wave. BALB/c mice were immunized with HA extracted (eHA) from H7N9 candidate vaccine viruses A/Shanghai/2/2013 (SH/2), A/Hong Kong/125/2017 (HK/125), or A/Guangdong/17SF003/2016 (GD/16) with or without adjuvant. Immunized mice were challenged with highly pathogenic avian influenza (HPAI) wild type (wt) GD/16 virus at 5 × 10^7^ TCID_50_/50 µl/mouse (high dose) in a BSL-3+ biocontainment. (**a**) IgG ELISA titers against GD/16 HA; (**b**) microneutralization (MN) titers against HPAI wt GD/16 virus; (**c**) Group 2 HA stem-specific IgG ELISA titers. (**d**) body weight (BW) and (**e**) survival after HPAI wt GD/16 challenge; (**f**) correlation of HA-specific IgG ELISA titers with survivals; (**g**) correlation of MN titers with survivals; (**h**) correlation of HA group 2 stem-specific IgG ELISA titers with survivals. Individual titers with geometric mean (bars) are shown (*n* = 5-15 mice/group). BW data are expressed as mean ± s.e.m (*n* = 5–10 mice/group). Correlation of antibody titers with mouse survival/mortality statuses after challenge was analyzed using logistic fit with χ2. Individual *p* values and *R*^2^ values are shown. Red dots indicate mortality (M) and blue asterisks indicate survival (S). *p < 0.05 and **p < 0.01 by Kruskal–Wallis test with Dunn’s multiple comparisons after log transformation (antibody responses) or by Log-rank (Mantel–Cox) test (survival curves).

We next compared antibody responses and protection induced by H7 eHAs against HPAI GD/16 challenges. The homologous prime-boost with 500 ng adjuvanted GD/16 eHA induced the highest GD/16-specific IgG ELISA and MN antibody titers in mice as compared to the other preparations tested (Fig. 3a, b). Mice immunized with GD/16 eHA, regardless of dose or adjuvant, had higher GD/16-specific IgG ELISA and MN GMTs than those immunized with SH/2 or HK/125 eHA at the same dose (Fig. 3a, b). Adjuvanted GD/16 or HK/125 eHA at 50 ng/dose showed an antigen-sparing effect by inducing higher GD/16-specific IgG GMTs than those elicited by unadjuvanted SH/2 eHA at 500 ng/dose (*p* < 0.01, Fig. 3a). Additionally, immunization with unadjuvanted or adjuvanted H7 eHAs induced HA group 2 stem-specific antibodies as measured by ELISA using a chimeric H6/H3 HA, with the highest titers observed in mice given 500 ng adjuvanted GD/16 eHA (Fig. 3c).

Following a high lethal dose HPAI challenge with wt GD/16 (5 × 10^7^ TCID_50_ /mouse), all immunization groups showed significant morbidity within the 1st week of infection. All mice from HK/125 or SH/2 eHA groups succumbed to death by 10 days post-infection (Fig. 3d). In contrast, 80% of mice immunized with adjuvanted 500 ng GD/16 eHA survived the highly lethal HAPI challenge and rapidly recovered weight (Fig. 3d, e). The group receiving 500 ng unadjuvanted GD/16 eHA had only 20% of mice survive the highly lethal HAPI challenge with slower recovery (Fig. 3d, e). We performed the regression analysis to correlate the survival/mortality statuses of individual mice in Fig. 3e, with their corresponding GD/16 and stem-specific IgG ELISA and MN titers by computationally fitting with χ2. The survival of individual mice after the highly lethal HPAI wt GD/16 challenge positively correlated with their GD/16 HA-specific IgG ELISA titers (*p* < 0.0001), higher HA-specific IgG titers and greater survival probability (Fig. 3f). Similarly, GD/16-specific MN titers positively correlated with surviving the highly lethal HPAI wt GD/16 infection (*p* < 0.0124, Fig. 3g). Moreover, HA group 2 stem-specific IgG ELISA titers showed a strong positive correlation with surviving the highly lethal HPAI wt GD/16 challenge (*p* < 0.0001, Fig. 3h). Mice with GD/16-specific IgG titer of 4.1 (Log10), MN titer of 2.8 (Log10), or stem-specific IgG titer of 4.2 (Log10) were predicted to have 50% chance surviving the highly lethal HPAI wt GD/16 challenge (Fig. 3f–h).

**Fig. 4:**
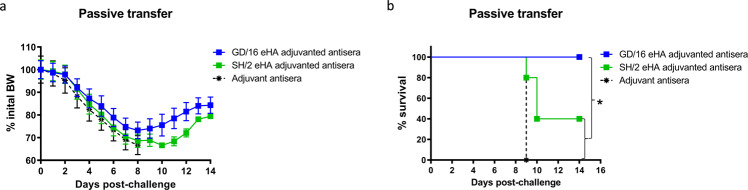
Passive transfer of hyperimmune sera against lethal H7N9 challenge. Hyperimmune sera were collected from mice immunized with HA extracted (eHA) from H7N9 A/Shanghai/2/2013 (SH/2) or A/Guangdong/17SF003/2016 (GD/16) at 500 ng/mouse with adjuvant. Pooled hyperimmune sera were injected intravenously into naïve mice followed by a lethal challenge of GD/16 IDCDC-56N virus. (**a**) Body weight (BW) and (**b**) survival after GD/16 IDCDC-56N challenge. BW data are expressed as mean ± s.e.m (*n* = 5 mice/group). *p < 0.05 by Log-rank (Mantel–Cox) test (survival curves).

In a passive antibody transfer experiment using GD/16 CVV as the challenge virus, all naïve mice receiving hyperimmune sera from adjuvanted GD/16 eHA-immunized mice survived lethal challenge despite initial BW loss (Fig. 4a, b). By contrast, naïve mice receiving adjuvanted SH/2 eHA-elicited hyperimmune sera or adjuvant-induced sera exhibited 60% or 100% mortality after both groups showed quick BW drop following the lethal challenge (Fig. 4a, b). These results, together with the LPAI challenge data show that first, antibodies induced by GD/16 eHA broadly protect against antigenically divergent H7N9 viruses and second, protection against HPAI H7N9 virus is based on its ability to elicit a more robust antibody response.

### Heterologous prime-boost immunization protects mice against lethal HPAI H7N9 challenges

**Fig. 5:**
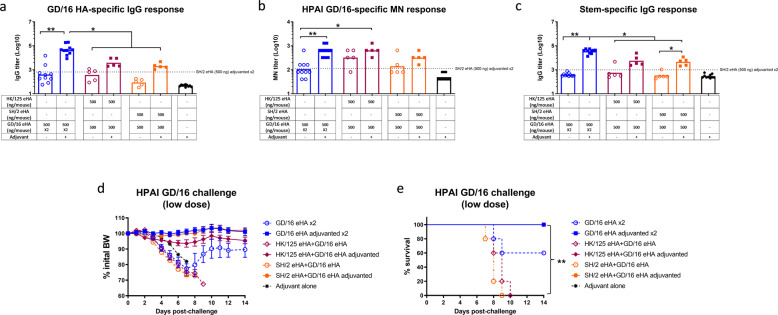
Immunogenicity and protection of heterologous prime-boost immunization against highly pathogenic avian influenza H7N9 challenge. BALB/c mice were primed with HA extracted (eHA) from H7N9 candidate vaccine viruses A/Shanghai/2/2013 (SH/2) or A/Hong Kong/125/2017 (HK/125) and boosted with A/Guangdong/17SF003/2016 (GD/16) with or without adjuvant. Immunized mice were challenged with highly pathogenic avian influenza (HPAI) wild type (wt) GD/16 virus at 5 × 10^4^ TCID_50_/50 µl/mouse (low dose) in a BSL-3+ biocontainment. (**a**) IgG ELISA titers against GD/16 HA; (**b**) microneutralization (MN) titers against HPAI wt GD/16 virus; (**c**) HA group 2 stem-specific IgG ELISA titers; (**d**) Body Weight (BW) and (**e**) survival after HPAI wt GD/16 challenge. Individual titers with geometric mean (bars) are shown (*n* = 5–10 mice/group). *p < 0.05 and **p < 0.01 by Kruskal–Wallis test with Dunn’s multiple comparisons after log transformation (antibody responses) or by Log-rank (Mantel–Cox) test (survival curves).

Because LPAI H7 eHAs (especially SH/2 eHA) are poorly immunogenic, we investigated whether heterologous prime-boost immunization regimen with GD/16 eHA enhances immunogenicity and protection against HPAI H7N9 viruses. As shown in Fig. 5a–c, mice receiving the heterologous prime-boost of adjuvanted HK/125 and GD/16 eHAs or adjuvanted SH/2 and GD/16 eHAs (all at 500 ng/dose unless otherwise specified) had GMTs (Log10) for GD/16 HA-specific IgG (3.6 or 3.3), GD/16-specific MN (2.8 or 2.5), or HA stem-specific IgG (3.7 and 3.6), respectively, higher than those induced by the homologous prime-boost of adjuvanted SH/2 eHA or unadjuvanted GD/16 eHA at the same dose. Homologous prime-boost with adjuvanted GD/16 eHA yielded the highest GMTs (Log10) of GD/16-specific IgG (4.6), MN (2.8,), and HA stem-specific IgG (4.5), respectively (Fig. 5a–c). Following a lower lethal challenge dose of HPAI wt GD/16 (5 × 10^4^ TCID_50_/mouse), mice with the homologous prime-boost of adjuvanted GD/16 eHA showed no morbidity and were fully protected (Fig. 5d, e). Mice receiving the heterologous prime-boost of adjuvanted eHAs (HK/125 and GD/16 or SH/2 and GD/16) also showed 100% survival without showing significant BW loss following the low lethal HPAI challenge (Fig. 5d, e). By contrast, all mice receiving the heterologous prime-boost of unadjuvanted eHAs or adjuvant succumbed to death with significant BW loss within 10 days of infection (Fig. 5d, e). Mice primed and boosted with unadjuvanted GD/16 eHA also lost approximately 20% BW but quickly recovered with 60% survival following the low lethal HPAI challenge (Fig. 5d, e).

These results show that inclusion of adjuvanted GD/16 eHA in heterologous prime-boost improves the immunogenicity and efficacy of LPAI H7 HAs against HPAI H7N9 virus in mice.

### Recombinant H7 HAs are immunogenic and protect mice against lethal HPAI H7N9 challenges

**Fig. 6:**
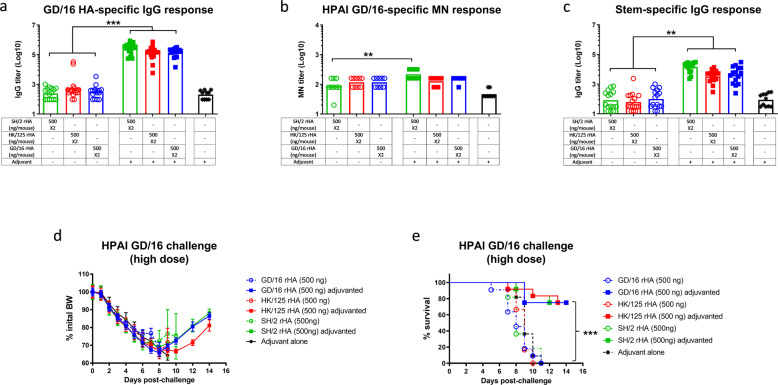
Immunogenicity and protection of recombinant HA against highly pathogenic avian influenza H7N9 challenge. BALB/c mice were immunized with recombinant HA (rHA) of H7N9 A/Shanghai/2/2013 (SH/2), A/Hong Kong/125/2017 (HK/125), or A/Guangdong/17SF003/2016 (GD/16) with or without adjuvant. Immunized mice were challenged with highly pathogenic avian influenza (HPAI) wild type (wt) GD/16 virus at 5 × 10^7^ TCID_50_/50 µl/mouse (high dose) in a BSL-3+ biocontainment. (**a**) IgG ELISA titers against GD/16 HA; (**b**) microneutralization (MN) titers against HPAI wt GD/16 virus; (**c**) HA group 2 stem-specific Ig titers; (**d**) Body Weight (BW) and (**e**) survival after HPAI wt GD/16 challenge. Individual titers with geometric mean (bars) are shown (*n* = 5–16 mice/group). **p < 0.01 and ***p < 0.001 by Kruskal–Wallis test with Dunn’s multiple comparisons after log transformation (antibody responses) or by Log-rank (Mantel–Cox) test (survival curves).

Previous studies reported that recombinant HA (rHA) expressed by the baculovirus/insect system shows good immunogenicity^14–16^. We next assessed the immunogenicity and protection of baculovirus-expressed H7 rHAs (Supplemental Fig. 2) in vivo. Similar to eHAs purified from H7 CVVs, two doses of adjuvanted rHAs at 500 ng/strain/mouse induced significantly higher antibody titers than those elicited by unadjuvanted rHAs at the same dose (Fig. 6a–c). Unlike SH/2 eHA, which was poorly immunogenic even with adjuvant, adjuvanted SH/2 rHA induced cross-reactive antibodies at similar levels to those elicited by adjuvanted GD/16 rHA (Fig. 6a–c). Similarly, administration of adjuvanted HK/125 rHA elicited comparable levels of cross-reactive antibodies in mice (Fig. 6a–c).

Despite initial BW loss after a highly lethal challenge with HPAI wt GD/16 (5 × 10^7^ TCID_50_ /mouse), 80% of mice immunized with adjuvanted GD/16, HK/125 or SH/2 rHAs survived and quickly recovered (Fig. 6d, e). In contrast, all mice from the groups of unadjuvanted rHAs died after significant morbidity (Fig. 6d, e). These results show that baculovirus-expressed H7 rHAs, especially SH/2 rHA, have improved immunogenicity and induce cross-protection against highly lethal HPAI H7N9 infection.

### Antibodies elicited by adjuvanted GD/16-derived HA target epitopes involved in H7N9 protection

**Fig. 7:**
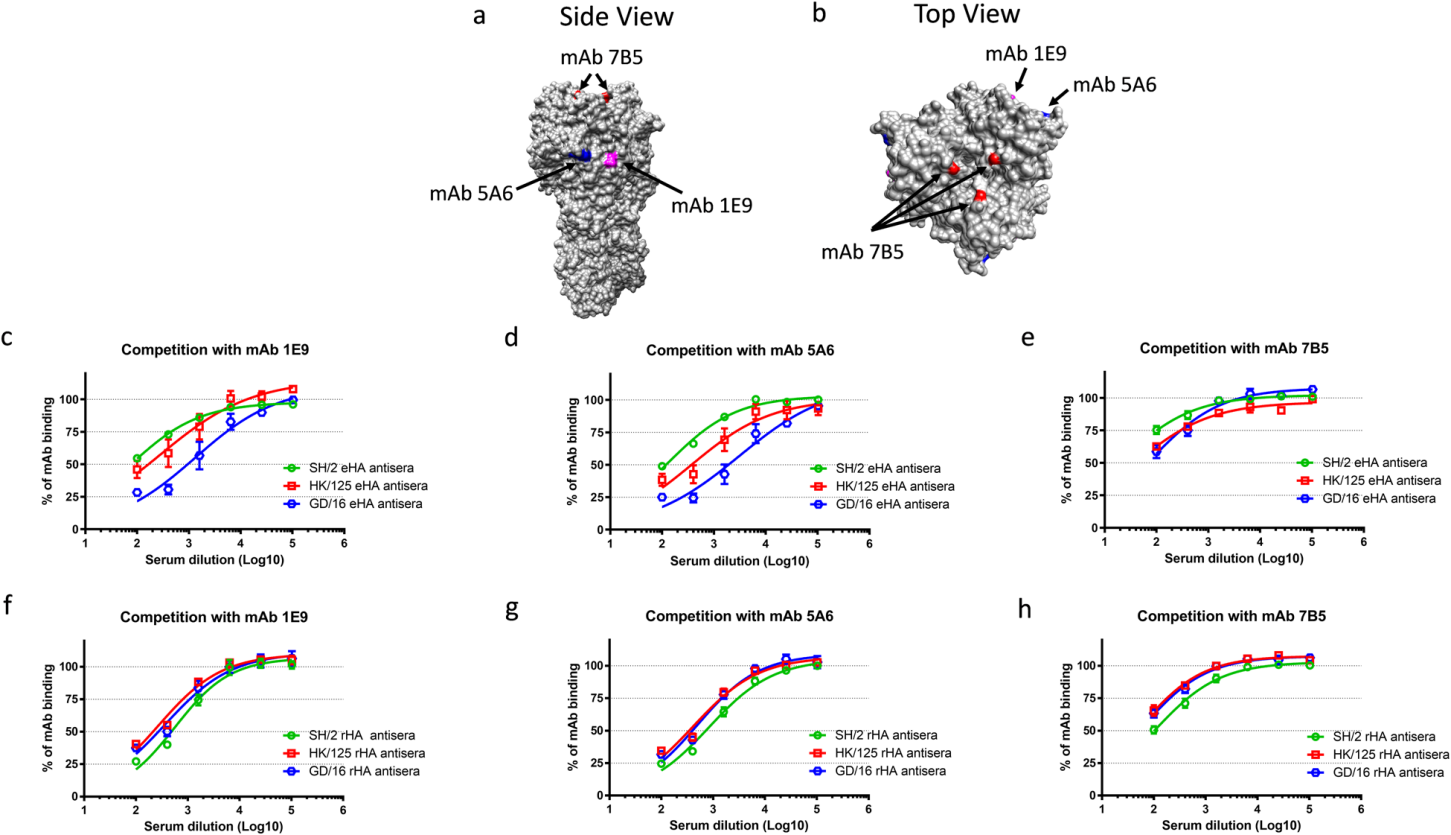
Competition of H7 HA-specific monoclonal antibodies with mouse hyperimmune sera. Hyperimmune sera were raised in mice administered with adjuvanted HA extracted (eHA) from H7N9 A/Shanghai/2/2013 (SH/2), A/Hong Kong/125/2017 (HK/125), or A/Guangdong/17SF003/2016 (GD/16) or H7 recombinant HAs (rHAs) at 500 ng/mouse. Hyperimmune sera were pooled and were used to compete with biotinylated H7-specific monoclonal antibody (mAb) 1E9, 5A6 or 7B5, respectively. The % of residual biotinylated mAbs as compared to the wells that contained only biotinylated H7 mAbs were plotted by nonlinear regression. (**a**) Side view and (**b**) top view of epitopes targeted by mAbs 1E9 (magenta), 5A6 (blue) and 7B5 (red) using SH/2 HA trimer (PDB ID: 4LN6) as the template; (**c**–**h**) competition with 1E9, 5A6 and 7B5. Data are expressed as mean ± s.e.m (*n* = 6–9 replicates/group).

To understand why immunization of adjuvanted GD/16 eHA induced broader cross-protection than that by adjuvanted SH/2 eHA, we conducted in vitro proliferation and ELISPOTs to assess cellular immunity elicited. The results showed that splenocytes harvested from mice immunized with adjuvanted SH/2 or GD/16 eHA exhibited comparable in vitro proliferation and produced similar levels of IgG, IL-4 or IFN-γ secreting spots after restimulation (Supplemental Fig. 3), indicating no detectable difference in B and T cell immunity induced. We then investigated if the quality of antibodies elicited by adjuvanted GD/16 eHA was different from that by adjuvanted SH/2 eHA by conducting competitive ELISA using biotinylated H7-specific monoclonal antibodies (mAbs). Neutralizing mAbs 1E9, 5A6, and 7B5 target distinct epitopes located in the globular head of H7 HA (Fig. 7a, b)^17,18^. Antibodies elicited by adjuvanted GD/16 eHA exhibited higher competitive capacity against mAb 1E9 than those induced by adjuvanted SH/2 or HK/125 eHA for HA binding (Fig. 7c). Antibodies elicited by adjuvanted GD/16 eHA also had the highest competitive capacity against mAb 5A6 followed by antibodies induced by adjuvanted HK/125 eHA and antibodies induced by adjuvanted SH/2 eHA were the least competitive against mAb 5A6 for HA binding (Fig. 7d). Antibodies elicited by all three adjuvanted eHA were equally competitive with mAb 7B5 for HA binding (Fig. 7e). Interestingly, antibodies elicited by adjuvanted SH/2 rHA showed enhanced HA binding and were equally competitive to antibodies elicited by adjuvanted GD/16 or HK/125 rHA against all three biotinylated mAbs (Fig. 7f–h).

These results indicate that immunization of adjuvanted GD/16 eHA resulted in broader antibody profile than those elicited by adjuvanted SH/2 or HK/125 eHA. Remarkably, immunization of baculovirus-expressed rHA, regardless of strain, yielded similar antibody binding profile for H7 HA.

## Discussion

The weak immunogenicity of H7N9 viruses has been noted since the first epidemic in 2013. Patients infected with SH/2-like H7N9 viruses had no H7-specific antibodies detected during the acute infection and had very low levels of H7-specific neutralizing antibodies (GMT40) appear in the convalescent phase^9^. The results from both pre-clinical and clinical studies show that unadjuvanted SH/2- or AH/1-based inactivated monovalent vaccines are poorly immunogenic, with a two-dose regimen required to achieve detectable antibody titers in immunized animals or human subjects^19–24^. Higher seroconversion and seroprotective rates were achieved after administration of two doses of adjuvanted vaccines containing higher contents of SH/2-like HA^19–24^. The immunogenicity studies of the fifth wave H7N9 viruses also showed that higher antibody responses were induced in animals immunized with two doses of adjuvanted inactivated whole viruses versus two doses of unadjuvanted or one dose of adjuvanted preparations^25,26^.

In the present study, we show that eHA extracted from HPAI GD/16 was more immunogenic than those extracted from LPAI SH/2 and HK/125 and induced higher and broader antibody responses against H7N9 viruses. The highest antibody titers were elicited in mice immunized with two doses of adjuvanted GD/16 eHA at 500 ng/mouse. This dose was considerably lower than those of inactivated SH/2-like monovalent vaccines evaluated in pre-clinical and clinical trials (3.7–45 µg HA/dose) or inactivated HK/125 or GD/16 whole virus vaccines tested in animal models (1–15 µg HA/dose)^19–26^. While inactivated split/subunit vaccines or whole virus-based preparations also contain other viral components such as neuraminidase that may play a role in protection^27,28^, we only focused on H7 HAs in the present study. Inclusion of AddaVax—a squalene-based adjuvant further reduced the required amount of GD/16 eHA per dose to 50 ng/mouse while still eliciting higher antibody responses than those induced by unadjuvanted eHAs at 500 ng/mouse. Such antigen-sparing and immune-enhancing effects have also been observed in the H7N9 vaccine clinical trials involving squalene-based (MF59 and AS03) or saponin-based (ISCOMATRIX) adjuvants^20–22,24,29^ but not aluminum hydroxide^23^. Higher antibody titers were seen in human subjects receiving two 3.75 µg/dose of inactivated SH/2-like monovalent vaccines adjuvanted with MF59 or AS03 versus those administered with two 45 µg/dose of unadjuvanted vaccines^22,24^. The antigen-sparing and immune-enhancing effects by adjuvanted vaccines are highly desired for pandemic preparedness, since it means less antigens are needed to achieve protective immunity and more doses of vaccines will be available during a pandemic outbreak from a given production run.

The present study also shows that mice primed and boosted with adjuvanted GD/16 eHA but not LPAI eHAs at 500 ng/mouse are protected from the lethal LPAI and HPAI H7N9 challenges. Protection appears to be mainly mediated by virus-specific antibodies, since passive transfer of GD/16-specific, but not SH/2-specific hyperimmune sera protected recipient mice from the lethal challenge of GD/16 CVV, consistent with a recent report that mice receiving hyperimmune sera raised by inactivated whole GD/16 CVV were protected from the challenges of GD/16 and AH/1 CVVs^26^. In the current study, we measured IgG ELISA and MN antibodies instead of HAI titers in mice following low doses of immunization, because H7N9 virus has high receptor binding avidity that greatly reduces HAI assay sensitivity^26^. MN assay detects not only antibodies blocking receptor binding but also HA stem, NA and M2 antibodies that mediate neutralization^30^. Thus, MN titer has been considered a more robust parameter than HAI titer for monitoring antibody responses to influenza viruses, especially those of avian origin^21,26,30,31^. Our study shows that mice with higher virus-specific MN titers were more likely to survive the lethal HPAI GD/16 challenge than those with lower MN titers. Higher virus-specific MN titers also correlated with lower viral burdens in the respiratory tract of ferrets immunized with adjuvanted SH/2-based monovalent vaccine followed by wild type AH/1 challenge^31^. In H7N9-infected patients, early induction of MN antibodies favors quick recovery from infection^32^.

In addition to MN titers, we also observed that both HA head- and stem-specific IgGs strongly correlated with the survival probability of mice challenged with HPAI wt GD/16 virus. In ferrets, H7-specific IgG ELISA titers were found to inversely correlate with respiratory virus loads after a LPAI H7N9 challenge^19^. Both HA head- and stem-specific IgGs were also found more sensitive than HAI or MN titers as surrogate markers of efficacy after a low immunogenic H7N1 vaccine failed to induce robust neutralizing antibodies in humans^33^. IgGs captured by quantitative ELISAs include both neutralizing and non-neutralizing antibodies. Neutralizing antibodies targeting the HA global head as those measured by HAI assay can prevent virus entry by blocking HA and host receptor interaction, while HA stem-specific neutralizing antibodies can inhibit pH-dependent conformational changes and interfere with membrane fusion to impair virus replication^34^. However, with few exceptions, HA head-specific neutralizing antibodies have limited cross-reactivity because the HA global head is highly variable across different subtypes. In contrast, the stem region is more conserved across different HA groups and neutralizing antibodies target the HA stem have the potential to confer broad cross-protection against influenza viruses^34,35^. In a household transmission study, HA stem-specific antibodies were founded to independently correlate with protection in humans infected with pandemic H1N1^28^. While neutralizing antibodies are better immune correlates of protection against influenza infections, non-neutralizing antibodies can also be protective by promoting viral clearance and reducing the disease severity via Fc-mediated effector functions (e.g. antibody-dependent cellular cytotoxicity)^31,36–38^. H7N9-specific non-neutralizing human monoclonal antibodies were found to protect recipient mice from a sublethal SH/2 challenge via FcγR engagement^39^.

Compared to adjuvanted SH/2 eHA, immunization of adjuvanted GD/16 eHA yielded better antibody quality by showing stronger competition with H7 mAbs for HA binding. 1E9, 5A6 and 7B5 are three neutralizing mAbs raised against SH/2 HA^17,18^. 1E9 and 5A6 recognize epitopes in the vicinity of HA antigenic site A, while 7B5 recognizes the epitope in antigenic site B, which is close to the receptor binding site^18^. Passive transfer of 5A6 protected recipient mice from SH/2 CVV challenge, while 7B5 and 1E9 were also found to recognize HAs of HK/125 and GD/16^18^. Thus, GD/16 HA-based vaccines may exercise cross-protection via both enhanced immunogenicity and improved antibody quality.

Moreover, we demonstrated that a heterologous prime-boost strategy could improve the performance of LPAI H7N9 CVVs, especially SH/2-based vaccines. Comparing the mice receiving homologous prime-boost immunization of adjuvanted SH/2 or HK/125 eHA, mice primed with adjuvanted SH/2 or HK/125 eHA followed by a heterologous booster of adjuvanted GD/16 eHA showed both enhanced immune responses and survivability after the lethal HPAI H7N9 challenge. This heterologous prime-boost strategy is important for the pandemic-influenza vaccine stockpile program, since currently tens of millions of doses of H7N9 vaccine, mostly SH/2-based, have been stockpiled by the US government for pandemic preparedness (https://www.medicalcountermeasures.gov/barda/influenza-and-emerging-infectious-diseases/pandemic-influenza-vaccine-stockpile-program/). By including GD/16 in the stockpile, we could make a good use of existing LPAI H7N9 vaccines and expand their protection coverage through the heterologous prime-boost strategy when the threat of an H7N9 pandemic looms. Additionally, we show that the recombinant approach significantly improved the immunogenicity and protective efficacy of LPAI SH/2 or HK/125 HA-based vaccines against HPAI H7N9 virus. Mice immunized with adjuvanted SH/2 or HK/125 rHA had virus-specific antibodies in similar level and quality to those administered with adjuvanted GD/16 rHA at 500 ng/mouse and were protected from the lethal HPAI wt GD/16 challenge. In humans, Flublok® based on baculovirus-expressed rHA was reported to elicit superior antibody responses than inactivated egg- or cell-based influenza vaccines^16^. Human antibodies elicited by AH/1 rHA vaccination were reported to broadly cross-react with emerging H7 HAs^15^. The improved immunogenicity and antibody quality were likely due to more stable conformational structure of rHA as compared to eHA contained in inactivated vaccines^14,19,40,41^. Previous studies have reported that the morphology of HA-containing structures affects the immunogenicity of influenza vaccines and inactivated split-virion or subunit vaccines containing large viral particles or spherical structures are generally more immunogenic than those (especially avian origin vaccines) containing small punctate structures^19,40,41^. The majority of baculovirus-expressed GD/16, SH/2 or HK/125 rHAs existed in 450-720 kDa oligomers that likely comprised 2-3 HA trimers (Supplemental Fig. 2). Under transmission electron microscopy, insect-expressed H7 rHA formed pleomorphic spheroid nanoparticles of approximately 20 nm with the size and morphology similar to those of Flublok® rHA vaccine^14^. Oligomeric HA has been reported to induce a higher antibody response than trimeric HA, while monomeric HA is the least immunogenic^42^.

In conclusion, we showed that two doses of HPAI H7N9 GD/16-derived HA with adjuvant-induced robust antibody response and protected mice against the lethal challenges of LPAI and HPAI H7N9 viruses from the first and fifth waves. These results demonstrated the immunogenicity and cross-protective efficacy of GD/16-based vaccine and provided strong evidence to support its inclusion in the influenza vaccine stockpile program for pandemic preparedness.

## Methods

### Viruses

LPAI H7N9 wt AH/1 and HK/125, and HPAI H7N9 wt GD/16 are select agents and had been stored and handled in a Select Agent Program regulated Biosafety Level (BSL)-3+ facility. All H7N9 CVVs bear a monobasic cleavage site in HA and have the internal genes replaced with those of A/PR/8/34 via reverse genetics. These H7N9 CVVs were handled under BSL-2 enhanced containment. All H7N9 viruses were propagated in 9–11 day old embryonated specific pathogen free (SPF) eggs at 37 °C. Allantoic fluids were aliquoted and stored at −70 °C until use. Infectivity as reflected by 50% tissue culture infectious dose (TCID_50_) were determined using a nucleoprotein-based ELISA^43^.

### H7 HA preparation

eHAs were extracted and purified from formalin-inactivated H7 CVVs following the procedures used to prepare antigens for raising standard reference antisera for vaccine potency determination^44^. Briefly, formalin-inactivated viruses were purified by sucrose gradient centrifugation and purified viruses (10 mg/ml) were incubated with 50 U/ml of bromelain (Sigma-Aldrich, Inc., St. Louis, MO) in TE buffer (10 mM Tris, 1 mM EDTA, pH 8.5) containing 50 mM beta-mercaptoethanol for 4 h at 37 °C with gentle shaking^44^. Following the first centrifugation at 30 000 rpm for 2 h at 4 °C, eHAs released in the supernatant was separated via 5–20% continuous sucrose gradients at 35,000 rpm for another 16 h at 10 °C. Purified eHAs were collected using Auto Densi-Flow Density Gradient Fractionators (Labconco, Kansas City, MO) and were verified with and without deglycosylation using PNGase F (New England Biolabs) (Supplemental Fig. 1).

H7 rHAs were designed to include a T4 foldon trimerization domain to ensure trimeric HA formation^45,46^. Briefly, the DNA sequences encoding HA ectodomain of GD/16 (without multibasic cleavage site), HK/125 or SH/2 with a T4 trimerization domain replacing the transmembrane domain and a 6xHis tag fused to the C-terminus were synthesized (GenScript, Piscataway, NJ)^45,46^. Group 2 chimeric rHA construct was also designed to include the globular head domain of an H6 HA (GenBank: AAO33485.1) atop of the stem region of an H3 HA (GenBank: AMB69181.1)^47,48^. Codon optimized DNA sequences were synthesized and were then subcloned into pFastBac1 vector for recombinant protein expression in baculovirus/insect cell system (GenScript). rHAs were purified by Ni-NTA affinity chromatography with ≥90% purity. Trimerized rHAs were verified with and without deglycosyaltion using PNGase F (Supplemental Fig. 2).

### Mouse immunization and challenge

SPF female adult BALB/c mice (Charles River laboratories, Frederick, MD) housed in the ABSL2 facility were primed and boosted intramuscularly with eHA or rHA alone or emulsified with AddaVax^TM^ (InvivoGen, San Diego, CA) at a 3-week interval. Sera were collected at 3 weeks after booster for antibody determination. Immunized mice were then transferred to the ABSL3 + facility for LPAI or HPAI wt H7N9 virus challenges. Under light isoflurane anesthesia, mice were intranasally inoculated with a low dose of wt AH/1, HK/125 or GD/16 at 5 × 10^4^ TCID_50_/50 µl/mouse, or a high dose of wt GD/16 at 5 × 10^7^ TCID_50_/50 µl/mouse in a BSL-3+ biocontainment. In some experiments, naïve mice were injected intravenously with 100 µl/mouse of pooled hyperimmune sera followed by a lethal challenge of GD/16 IDCDC-56N (5 × 10^7^ TCID_50_/50 µl/mouse) at BSL-2. BW and mortality were monitored daily for two weeks after challenges. Mice reaching humane endpoints (e.g. 30% BW loss) were immediately euthanized. All procedures were performed according to the animal study protocols approved by the FDA White Oak Animal Program Animal Care and Use Committee.

### IgG ELISA

Sera were pre-treated with receptor-destroying enzyme (Denka-Seiken, Tokyo, Japan) followed by heat-inactivation before antibody assessment. IgG ELISA was performed in 96-well microtiter plates pre-coated with 0.5 µg/ml of H7 rHAs^43^. Bound antibodies were detected using peroxidase-conjugated secondary antibodies (Life technologies, Frederick, MD) followed by TMB substrate. Optical density (OD) at 450 nm was measured using a Victor V multilabel reader (PerkinElmer, Waltham, MA). IgG ELISA titers were interpolated based on a standard curve constructed using mouse hyperimmune sera collected after HPAI H7N9 challenge.

### Competitive ELISA

H7-specific mAbs 1E9, 5A6 and 7B5^18^ were biotinylated at 1:1 molecular ratio using iLink™ biotin antibody labeling kit (ABP Biosciences, Rockville, MD). Serially diluted mouse sera were incubated in rHA pre-coated 96-well microtiter plates at room temperature for 2 h followed by thoroughly washing. Biotinylated H7 mAbs were then added and were incubate for another 1 h. Residual biotinylated mAbs after thoroughly washing were detected using peroxidase-conjugated streptavidin (Life technologies) as described above. The % of residual biotinylated mAbs as compared to the wells that contained only biotinylated H7 mAbs were plotted by nonlinear regression using Prism 6 (GraphPad, San Diego, CA).

### Microneutralization (MN) assay

A cell-based MN assay was performed^43^ with minor modifications. Briefly, 100 TCID_50_ of wt LPAI AH/1, HK/125 or HPAI GD/16 were incubated with RDE-treated sera at room temperature for 1 h. The virus-serum mixtures were then incubated with Madin-Darby Canine Kidney cells (2 × 10^4^ cells/well) in 96-well tissue culture plates at 37 °C, 5% CO2 for 5 days. Cells with cytopathic effect were detected by staining with 1% crystal violet solution. MN titers represent the reciprocal of the highest serum dilution resulting in 100% cell viability.

### ELISPOT

Mouse IgG, IFN-ɣ or IL-4 ELISPOT was performed using Mabtech’s ELISPOT BASIC kits (Cincinnati, OH) as previously reported^49^ with minor modifications. For IgG ELISPOT, splenocytes were pre-activated with R848 (1 µg/ml) and mIL-2 (10 ng/ml) at 37 °C for 3 days before being added to rHA (15 µg/ml) pre-coated 96-well Multiscreen filter ELISPOT plates (EMD Millipore, Billerica, Massachusetts). For IFN-ɣ or IL-4 ELISPOT, splenocytes were added to anti-IFN-ɣ or anti-IL-4 pre-coated ELISPOT plates and were restimulated with purified H7 CVVs (10 µg/ml) at 37 °C for 72 h. Cells incubated with medium or PMA/IM mixture (2 µg/ml PMA plus 2 µM IM) served as negative or positive controls. Antigen-specific spot-forming units were detected using biotinylated secondary antibodies and were counted using the AID vSpot Spectrum (Autoimmun Diagnostika GmbH, Strassberg, Germany). The number of antigen-specific spots were expressed as number of spots per 10^6^ cells after subtracting the number of spots in unstimulated negative controls. Each mouse sample was assayed in triplicates.

### Cell proliferation

Splenocyte proliferation was determined using a Bromodeoxyuridine (BrdU)-based ELISA kit (Roche, Indianapolis, Indiana). Dissociated splenocytes were incubated with purified H7 CVVs (20 µg/ml) or PMA/IM mixture (2 µg/ml PMA plus 2 µM IM) in black 96-well ViewPlates (Perkin Elmer, Waltham, Massachusetts) at 37 °C for 90 h^49^. After incubation with BrdU labeling solution for additional 2 h, cells were fixed and incorporated BrdU was detected using peroxidase-conjugated anti-BrdU monoclonal antibody according to the manufacturer’s instructions. Cell proliferation was expressed as fold induction vs the absorbance of unstimulated control cells at 450 nm. Each mouse sample was assayed in triplicates.

### Epitope modeling

The epitopes targeted by 1E9, 5A6 and 7B5 mAbs were mapped in the structure of SH/2 HA trimer (PDB ID: 4LN6) using Chimera (http://www.rbvi.ucsf.edu/chimera)^50^.

### Statistical analysis

Antibody titers were log transformed before being subjected to nonparametric test (Kruskal-Wallis test with Dunn’s multiple comparisons) using Prism 6 (GraphPad). The correlation between virus-specific antibody titers and mouse survival/mortality status after challenge was analyzed using logistic fit with χ^2^ (JMP statistical software, version 14). A *p* value of < 0.05 was considered statistically significant.

### Reporting summary

Further information on research design is available in the Nature Research Reporting Summary linked to this article.

## Supplementary information

Supplementary Information

Reporting Summary

## Data Availability

All the relevant information is in the article and supplementary material. The unique materials/reagents used in this study are available from the corresponding author upon reasonable request and by Material Transfer Agreement.
