# Supplementary material for: Highly pathogenic avian influenza A/Guangdong/17SF003/2016 is immunogenic and induces cross-protection against antigenically divergent H7N9 viruses
Source: NPJ Vaccines. 2021 Feb 26;6:30. doi: 10.1038/s41541-021-00295-7 (PMC7910538; doi:10.1038/s41541-021-00295-7)
Supplement: Supplementary file 1 — Supplementary Information [file 41541_2021_295_MOESM1_ESM.pdf]

Highly pathogenic avian influenza A/Guangdong/17SF003/2016 of the 5th wave is immunogenic and induces broad cross-protection against antigenically divergent H7N9 viruses

Peter Radvak[1], Martina Kosikova[1], Yuan-Chia Kuo[1], Xing Li[1], Richard Garner[1], Falko Schmeisser[2], Ivan Kosik[3], Zhiping Ye[1], Jerry P. Weir[2], Jonathan W. Yewdell[3], Hang Xie[1*]

[1]Laboratory of Pediatric and Respiratory Viral Diseases, [2]Laboratory of DNA Viruses, Division of Viral Products, Office of Vaccines Research and Review, Center for Biologics Evaluation and Research, United States Food and Drug Administration, Silver Spring, Maryland, USA; [3]Laboratory of Viral Diseases, National Institute of Allergy and Infectious Diseases, National Institutes of Health, Bethesda, Maryland, USA.

Supplemental figures and legends

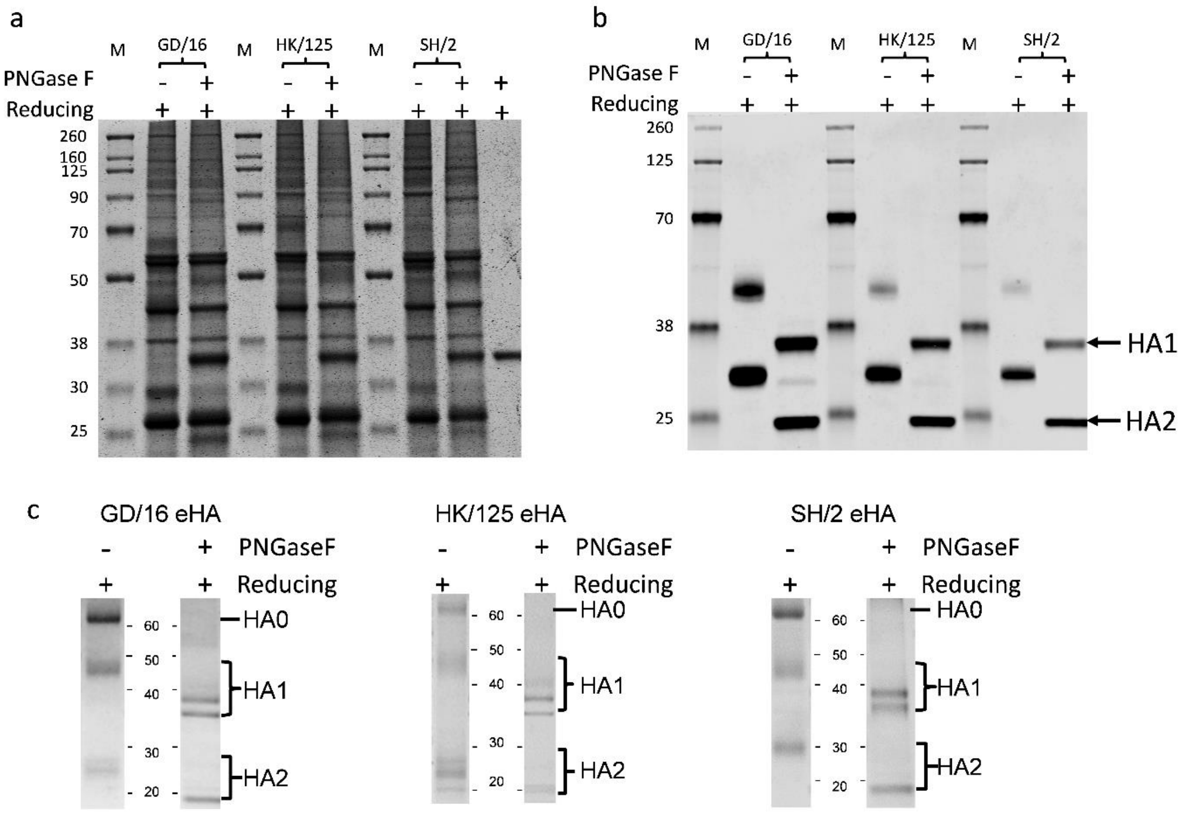

Supplemental Figure 1. SDS-PAGE analysis of HA extracted from H7N9 candidate vaccine virus. HAs extracted (eHAs) from H7N9 candidate vaccine viruses (CVVs) A/Guangdong/17SF003/2016 (GD/16), A/Hong Kong/125/2017 (HK/125) or A/Shanghai/2/2013 (SH/2) were purified. (a) H7N9 CVVs with or without deglycosylation using PNGase F; (b) HA-specific western blot of H7N9 CVVs; (c) Purified H7 eHAs with or without deglycosylation using PNGase F.

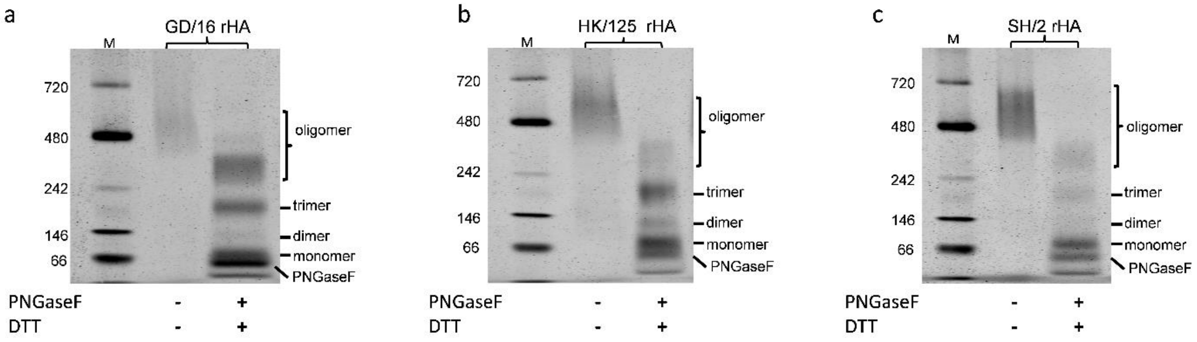

Supplemental Figure 2. Native PAGE analysis of Recombinant H7 HA. Recombinant HA (rHA) from H7N9 viruses (a) A/Guangdong/17SF003/2016 (GD/16), (b) A/Hong Kong/125/2017 (HK/125) or (c) A/Shanghai/2/2013 (SH/2) was subjected to native PAGE with or without deglycosylation using PNGase F.

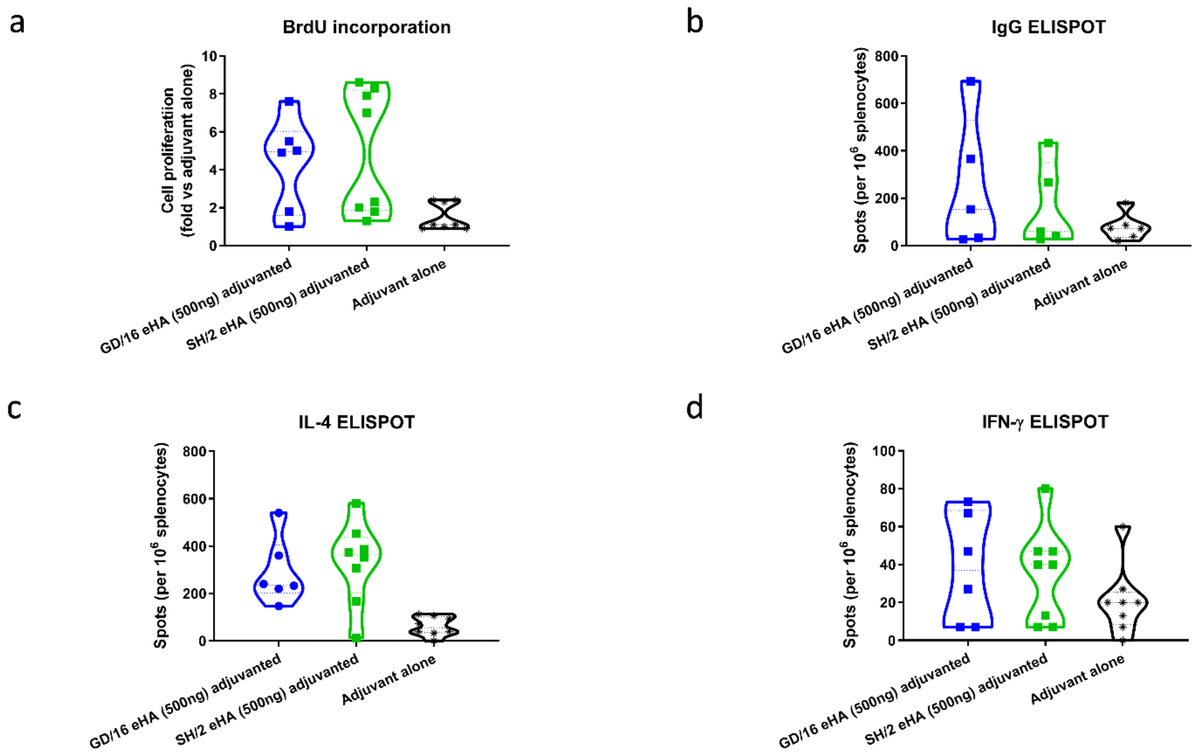

Supplemental Figure 3. In vitro proliferation and ELISPOT after immunization. Splenocytes were harvested from mice immunized with adjuvanted HA extracted (eHAs) from H7N9 A/Shanghai/2/2013 (SH/2), A/Hong Kong/125/2017 (HK/125), or A/Guangdong/17SF003/2016 (GD/16) and were restimulated in vitro with purified H7N9 candidate vaccine viruses or recombinant HAs (rHAs) for proliferation and ELISPOTs. (a) Cell proliferation; (b) IgG ELISPOT; (c) IL-4 ELISPOT; (d) IFN-γ ELISPOT. n=5-8 replicates/group.
