# Supplementary material for: Highly pathogenic avian influenza A/Guangdong/17SF003/2016 is immunogenic and induces cross-protection against antigenically divergent H7N9 viruses
Source: NPJ Vaccines. 2021 Feb 26;6:30. doi: 10.1038/s41541-021-00295-7 (PMC7910538; doi:10.1038/s41541-021-00295-7)
Supplement: Supplementary file 2 — Reporting Summary [file 41541_2021_295_MOESM2_ESM.pdf]

nature research

Corresponding author(s): Hang Xie
Last updated by author(s): Jan 30, 2021

Reporting Summary

Nature Research wishes to improve the reproducibility of the work that we publish. This form provides structure for consistency and transparency in reporting. For further information on Nature Research policies, see our Editorial Policies and the Editorial Policy Checklist.

Statistics

For all statistical analyses, confirm that the following items are present in the figure legend, table legend, main text, or Methods section.

n/a	Confirmed	
☐	☒	The exact sample size (*n*) for each experimental group/condition, given as a discrete number and unit of measurement
☐	☒	A statement on whether measurements were taken from distinct samples or whether the same sample was measured repeatedly
☐	☒	The statistical test(s) used AND whether they are one- or two-sided<br>*Only common tests should be described solely by name; describe more complex techniques in the Methods section.*
☒	☐	A description of all covariates tested
☒	☐	A description of any assumptions or corrections, such as tests of normality and adjustment for multiple comparisons
☐	☒	A full description of the statistical parameters including central tendency (e.g. means) or other basic estimates (e.g. regression coefficient) AND variation (e.g. standard deviation) or associated estimates of uncertainty (e.g. confidence intervals)
☒	☐	For null hypothesis testing, the test statistic (e.g. *F*, *t*, *r*) with confidence intervals, effect sizes, degrees of freedom and *P* value noted<br>*Give P values as exact values whenever suitable.*
☒	☐	For Bayesian analysis, information on the choice of priors and Markov chain Monte Carlo settings
☒	☐	For hierarchical and complex designs, identification of the appropriate level for tests and full reporting of outcomes
☒	☐	Estimates of effect sizes (e.g. Cohen's *d*, Pearson's *r*), indicating how they were calculated

Our web collection on statistics for biologists contains articles on many of the points above.

Software and code

Policy information about availability of computer code

Data collection	Data were collected using Microsoft Excel or built-in software in Victor V multilabel reader (PerkinElmer) & AID vSpot Spectrum (Autoimmun Diagnostika GmbH).
Data analysis	Microsoft Excel; GraphPad Prism 6; JMP version 14; Chimera (http://www.rbvi.ucsf.edu/chimera)

For manuscripts utilizing custom algorithms or software that are central to the research but not yet described in published literature, software must be made available to editors and reviewers. We strongly encourage code deposition in a community repository (e.g. GitHub). See the Nature Research guidelines for submitting code & software for further information.

Data

Policy information about availability of data

All manuscripts must include a data availability statement. This statement should provide the following information, where applicable:

- Accession codes, unique identifiers, or web links for publicly available datasets
- A list of figures that have associated raw data
- A description of any restrictions on data availability

All the relevant information is in the article and supplementary material.

Field-specific reporting

Please select the one below that is the best fit for your research. If you are not sure, read the appropriate sections before making your selection.

☒ Life sciences ☐ Behavioural & social sciences ☐ Ecological, evolutionary & environmental sciences

For a reference copy of the document with all sections, see nature.com/documents/nr-reporting-summary-flat.pdf

Life sciences study design

All studies must disclose on these points even when the disclosure is negative.

Sample size	In general 5-10 mice per group were used, which was consistent with similar studies as published by other labs. The sample size for each experiment is indicated in the figure legends.
Data exclusions	No data were excluded
Replication	All vitro assays were performed in triplicates. All attempts at replication were successful.
Randomization	The animals were randomly grouped and allocated in each experiment.
Blinding	Not applicable.

Reporting for specific materials, systems and methods

We require information from authors about some types of materials, experimental systems and methods used in many studies. Here, indicate whether each material, system or method listed is relevant to your study. If you are not sure if a list item applies to your research, read the appropriate section before selecting a response.

Materials & experimental systems

n/a	Involved in the study	
☐	☒	Antibodies
☐	☒	Eukaryotic cell lines
☒	☐	Palaeontology and archaeology
☐	☒	Animals and other organisms
☒	☐	Human research participants
☒	☐	Clinical data
☒	☐	Dual use research of concern

Methods

n/a	Involved in the study	
☒	☐	ChIP-seq
☒	☐	Flow cytometry
☒	☐	MRI-based neuroimaging

Antibodies

Antibodies used	Goat anti-Mouse IgG (H+L) Secondary Antibody, HRP (Invitrogen cat no 31430);<br>Streptavidin-Horseradish Peroxidase (HRP) Conjugate (Invitrogen cat no SA10001);<br>The H7-specific mAb 1E9, 5A6 and 7B5 were produced in house and relevant references were provided in the main text.
Validation	The validation of commercial antibodies can be found from the manufacturers' websites.<br>The H7-specific mAb 1E9, 5A6 and 7B5 were characterized in detail before and relevant references were provided in the main text.

Eukaryotic cell lines

Policy information about cell lines

Cell line source(s)	Madin-Darby Canine Kidney (MDCK) cell line was from ATCC
Authentication	MDCK is commercially available. Only passages were performed after MDCK was received and recovered.
Mycoplasma contamination	MDCK was negative for mycoplasma contamination.
Commonly misidentified lines<br>(See ICLAC register)	No commonly misidentified cell lines were used.

Animals and other organisms

Policy information about studies involving animals; ARRIVE guidelines recommended for reporting animal research

Laboratory animals	SPF female 4-5 week-old BALB/c mice were purchased from Charles River laboratories, Frederick, MD.
Wild animals	No wild animals were used.
Field-collected samples	No field-collected samples.
Ethics oversight	The animal study protocol was approved by the FDA White Oak Animal Program Animal Care and Use Committee

Note that full information on the approval of the study protocol must also be provided in the manuscript.
